# A study and training of careers on effective cognitive therapy for elderly to prevent dementia in Lagos, Nigeria. (Proposed Project)

**DOI:** 10.1002/alz.092560

**Published:** 2025-01-09

**Authors:** Feyikemi Mary Akinyelure, Tolulope Adeyoola, Adetayo Adetutu Adeife, Lucky Jet

**Affiliations:** ^1^ Dora‐Care Behavioral Foundation., Lagos, Lagos Nigeria; ^2^ Federal Neuropsychiatric Hospital, Yaba, Lagos, Yaba, Lagos Nigeria; ^3^ Federal Neuropsychiatric Hospital, Yaba, Lagos., Yaba, Lagos Nigeria

## Abstract

**Background:**

The immediate family members, trusted caregivers or the society often subject the elderly in Nigeria to abuse. The most vulnerable amongst the elderly are poor and disadvantaged ones who often times are on the receiving end. Effective cognitive training is a non‐pharmacology treatment that focuses on guided practice on cognitive tasks.

**AIMS:**

To train careers in effective cognitive therapy (graded table top activities, memory exercises activities, problem solving games) and activities of daily routine.

To determine the effectiveness of cognitive therapy game impact on older adult in preventing Dementia, Alzheimer e.t, c.

To evaluate the feasibility of the training intervention implementation.

**Methods:**

We'll train 120 health care assistant annually in Lagos Nigeria.

Training: Theoretical (Dementia knowledge), Practical (Dementia assessment and screening)

Evaluation: Standardized Mini Mental‐State Examination and the clock drawing test (CDT)

Expected outcomes.

The primary outcome is to train careers in caring for the elderly effectively which would lead to prolong life.

The secondary outcome is to reduce the impact of dementia through knowledge and skills the careers must have gained.

